# Regional Odontodysplasia: Report of a case

**Published:** 2013-12

**Authors:** A Rashidian, M Afsharian Zadeh, M Azarshab, T Zarrabian

**Affiliations:** a Dept. of Pediatrics, Dental Branch, Islamic Azad University, Tehran, Iran; b Pediatric Dentist

**Keywords:** Regional Odontodysplasia, Ghost Teeth, Odontodysplasia

## Abstract

Regional odontodysplasia is a rare dental anomaly affecting both primary and adult dentitions in the maxilla or mandible or both jaws, whilst involvement of the maxilla is more common [1-2]. In most cases, one quadrant is affected. One of the characteristics of this anomaly is discolored and soft teeth that can be accompanied by swelling or abscess. In this anomaly, enamel and dentin are thin and hypoplastic, therefore, the teeth give the impression of “ghost teeth” [2, 8]. In radiography, the delineation between enamel and dentin is not clear and pulp chamber is wide. Histologically, areas of hypocalcified enamel are observed and the enamel prisms appear to be irregular in direction [2]. There is a disturbance in dentin formation and dentinal tubules are reduced in number. The etiology of regional odontodysplasia is still unknown [8]. Managements of these cases should be based on the esthetics and functional needs as well as the degree of involvement.

This report describes a case of regional odontodysplasia in a 3.5 year old Iranian girl whose chief complaint was the abscess formation in the left maxillary primary molar region. This case study aims to report the clinical and radiological findings of the current case.

## Introduction

The condition was first described by Hitchin in 1934 [8], although the first report was published by McCall et al. in 1947 [7]; in which the condition was named “arrested tooth development”. In 1954, Rushton introduced the term “shell teeth” to portray the radiological characteristic of this anomaly. In 1963, Zegarelli et al, proposed the term “odontodysplasia” and in 1970, Pindborg added the “regional” to include that this condition involves a region or a segment of a jaw [9]. 

Regional odontodysplasia is a rare developmental dental anomaly and the prevalence of this condition is still not definitely clear since the studies have mainly been based on the case reports [1-5]. Its prevalence is reported to be less than 1/1000000 and only about 140 cases have been reported in the literature up to the time of this study [6]. This anomaly affects dental hard tissues of both ectodermal and mesodermal origin [7]. 

The etiology of regional odontodysplasia is unknown, but several factors such as local trauma, infection, ischemia or vascular defects, neural damage, hyperpyrexia, nutritional deficiencies, teratogenic drugs, Rh incompatibility, activation of latent viruses residing in odontogenic epithelium, somatic mutations and disorders of neural crest cell migration have been advocated [9-11]. It reported that regional odontodysplasia is probably a non-hereditary condition [11]. 

The regional odontodysplasia has no racial predilection and affects female more than male individuals (ratio F/M = 1.4/1) [9]. Although this condition mostly affects one quadrant; 7 cases of generalized odontodysplasia have been reported in the literature [11-12]. 

The regional odontodysplasia can occur in maxilla or mandible or both, although involvement of maxilla is twice and the involvement is usually unilateral. One of the distinguishing characteristics of regional odontodysplasia is that the condition rarely crosses the midline [9]. 

The affected teeth are clinically small, grooved, hypoplastic and hypocalcified [13]. The involved enamels are soft on probing and the implicated teeth are susceptible to caries [7]. 

These teeth often display a brown or yellowish discoloration. The most frequent clinical symptoms accompanied by this anomaly are failure of eruption, swelling or abscess of gingiva and periapical infection. Moreover, nine cases of hemangioma and six cases with facial hemi-atrophy were reported to be present with regional odontodysplasia [11]. 

Radiologically, the affected teeth illustrate abnormal morphology and hypoplastic crown and the lack of contrast between enamel and dentin is usually apparent. The enamel and the dentin are very thin, displaying a” ghost-like appearance” [7]. The enlarged pulp chambers, short roots, open apices and shell-like crowns are the other pathognomic radiological characteristics [7]. 

In microscopic examination, reduction in the thickness of the dentin layer, expanded areas of inter-globular dentin and extended pre-dentin is observed.

 In severe cases, the dentinal tubules are reduced in number, whereas in the milder cases the mantle dentin appears normal. The pulp often contains denticles and amorphous calcified materials [11]. 

The purpose of this study is to report a case of regional odontodysplasia in a 3.5-year-old Iranian girl and thereby describing its clinical and radiographic features.

## Case Report

A 3.5-year-old girl was referred to the Department of Pediatric Dentistry, Dental School, Islamic Azad University; Tehran, Iran. Her chief complaint was the abscess formation in the left maxillary region. The prenatal, natal conditions of the patient and the attained medical history revealed no explicit findings. Both parents did not report any previous history of tooth abnormalities or genetic anomalies on either maternal or paternal family part of the case. There was negative history concerning the delayed eruption of the maxillary left teeth compared to their counterparts. Based on her mother’s report, the abscess existed from six month ago and its size was reduced after administration of antibiotics.

The extra-oral examination revealed no swelling, no facial asymmetry and the lymph nodes were not palpable. 

The intra-oral examination showed the occlusion type was mesial step on both sides and the oral hygiene was excellent. Oral mucosa was normal, except for an abscess on the gingiva above the maxillary left primary molar; which was fluctuant and about 0.5 x o.5 cm^2^ in size.

**Figure 1a:**
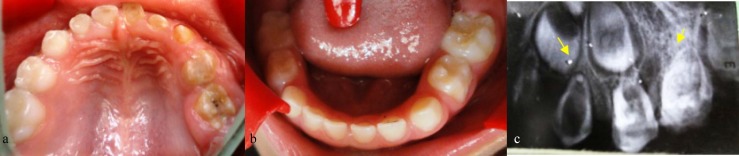
Occlusal view of maxillary arch, all primary teeth in left maxilla are affected **b** Occlusal view of mandibular arch: all mandibular teeth have normal size and are caries-free **c **Periapical view of maxillary left primary canines and molars. Note the ghost teeth in the radiograph

All the primary teeth on the left quadrant of the maxilla had abnormal crown, with a yellowish discoloration and hypoplastic enamel. The right maxillary central and lateral incisors as well as the canine had minor caries. The first primary molar was considerably hypoplastic with an obvious carious lesion. The abscess was considered to be related to the maxillary left first primary molar. The right maxillary and mandibular teeth were normal and caries-free (Figure 1a, b).

The periapical (Figure 1c) and the panoramic radiographs were taken and the radiological images showed that the mandibular and the right maxillary teeth were normal. All the teeth in the left maxillary region were present, although they displayed a typical “ghost-like” appearance in the radiographs. The demarcation line between the enamel and the dentin of the affected teeth was not clear and the enamel was hypoplastic. All the involved teeth exhibited enlarged pulp chambers and short-slender roots. A periapical radiolucent lesion was detected in the maxillary left primary molar region which was related to the gingival abscess. The maxillary left first permanent molar appeared to suffer from the same anomaly.

The laboratory examinations showed that the serum sodium, potassium, calcium, phosphorous and alkaline phosphatase levels were in the normal range. 

On the basis of our clinical and radiological findings, a diagnosis of regional odontodysplasia was proposed. The patient was instructed for extraction of the left maxillary primary molar, restoration of the maxillary second molar with stainless steel crown (SS-crown) and a space maintainer (crown, band and loop) to maintain the space after extraction. Moreover, a glass ionomer build-up restoration for the left maxillary incisors and canine was deliberated. A programmed follow-up visits was planned for maxillary left permanent molar to be built-up by glass ionomer restorations as soon as completion of its eruption.

## Discussion

The regional odontodysplasia is a relatively rare developmental anomaly [1-6]; therefore, the report of this case seems to be imperative. The condition affects the dentin and enamel of a group of adjacent teeth. This case presents several common clinical and radiological features related to the regional odontodysplasia. According to the Lustmann et al. [14], teeth in the maxillary arch are affected more frequently by regional odontodysplasia and this anomaly rarely crosses the midline. The patient in this report had “ghost teeth” in the maxillary left region that ended exactly in the midline. Based on the literature, regional odontodysplasia is more prevalent in females, and our case was also a girl. The chief complaint was an abscess formation of the maxillary left primary molar in accordance with other reports [4, 7-11]. Although several authors reported failure or delayed eruption of the affected teeth [13]; we did not find any problem regarding this issue. 

The etiology of regional odontodysplasia has not been clearly understood. However, many theories are proposed concerning its etiology [10] such as nutritional deficiencies, local trauma, infection, ischemia or vascular defects, neural damage, hyperpyrexia, teratogenic medications and facial nevi [10]. In this case, however, none of these factors could be related to the regional odontodysplasia, as there were no history of using medications during mother’s pregnancy, any nutritional deficiency or allergy to milk, and there were no signs of facial nevi or vascular/ neural defects.

The management of a child with regional odontodysplasia entails a multidisciplinary approach. The goal of the treatment is to improve the esthetics and to provide a good oral and dental function for the involved child [15]. There is no consensus whether affected teeth (with or without abscess) should be kept or extracted [10]. Some clinicians believe that extracting the affected teeth and inserting a prosthetic appliance is the best treatment, although others advise for a restorative approach. It transpires that in young children, affected teeth should be kept and the teeth associated with abscess might not be restored and consequently should be extracted [10]. 

In the current case, the maxillary left primary molar should be extracted. The remaining carious teeth in the left quadrant can be restored. The best modality is to restore the left maxillary second molar with stainless steel crown and likewise, the resin-modified glass ionomer build-up restoration for left maxillary incisors and canine would be the best choice. A space maintainer would also be desirable following the extraction of the maxillary left primary molar.

Programmed follow-up examinations should be considered to observe and control the eruption of the maxillary left first permanent molar to perform fissure-sealant treatment as soon as it erupts.
